# Insights into the changes of soil microorganism communities and carbon cycle metabolic functions caused by the application of L-glufosinate-ammonium

**DOI:** 10.3389/fmicb.2026.1920057

**Published:** 2026-08-26

**Authors:** Yuanfeng Dai, Han Li, Hancheng Wang, Yi He, Kai Cai, Xiaojun Wu, Huaihao Zou, Ying Wang, Liuti Cai, Xiaomao Wu, Imran Haider Shamsi

**Affiliations:** 1 Bijie Tobacco Company, Bijie, China; 2 Guizhou Provincial Academician Workstation of Microbiology and Health, Guizhou Academy of Tobacco Science, Guiyang, China; 3 College of Agriculture, Guizhou University, Guiyang, Guizhou, China; 4 Anshun Tobacco Company, Anshun, China; 5 Department of Agronomy, College of Agriculture and Biotechnology, Zhejiang University, Hangzhou, China

**Keywords:** carbon cycle, correlation, L-glufosinate-ammonium, metabolomics, microbial communities, soil properties

## Abstract

L-glufosinate-ammonium (L-GLA), a widely used herbicide, exerts detrimental non-target effects on crops, soil microorganisms, and ecosystems. However, its impacts on soil microbial communities and metabolic functions remain poorly understood. In this study, we applied L-GLA at two concentrations—600 g a.i. hm^−2^ (low, L) and 3,000 g a.i. hm^−2^ (high, H)—to yellow-brown soil and investigated its effects on microbial community composition, carbon cycle-related metabolic functions, and soil metabolites using integrated metagenomics and soil environmental pseudotargeted metabolomics at 30 and 60 days post-application. The degradation rate of L-GLA was concentration-dependent, with half-lives of 18.8 days (L) and 29.7 days (H). Both doses significantly reduced soil organic matter (SOM) and available potassium (AK) content, and markedly altered microbial community richness, structure, and composition. L-GLA exposure also disrupted the complexity of soil microbial co-occurrence networks and the activities of carbon-cycle-related enzymes. Metabolomic analysis further revealed significant (*p* < 0.05) and dose-dependent alterations in the soil metabolite profile. Correlation analysis indicated strong associations between characteristic microbial taxa and differential metabolites. Our findings provided critical insights into how L-GLA influences the soil microecological environment and contributed to a deeper understanding of soil microbial ecology in the context of modern agricultural practices.

## 1 Introduction

Glufosinate, a widely used broad-spectrum contact herbicide,plays a key role in the herbicide-resistant crop cultivation system. It is widely referred to as glufosinate-ammonium, this is due to the widespread use of its ammonium-salt formulations (Takano and Dayan, 2020). Commercial application is mainly based on racemic glufosinate-ammonium (GLA) (Composed of two enantiomers, L-GLA and D-glufosinate-ammonium, in a 1:1 ratio) (Garrison et al., 2011; Feng et al., 2023). However, there are essential differences in the biological activity between the two isomers: L-GLA is an isomer with herbicidal activity, with a herbicidal efficiency that can reach 8–10 times that of inactive D-glufosinate-ammonium (Mao et al., 2014; Zhang L. et al., 2019), and a lower application dose under the same control effect; theoretically, it has the application advantage of “high efficiency and low dosage” (Yue et al., 2020). With the increasing demand for “low toxicity and high efficiency” pesticides in green agriculture, L-GLA has gradually become a focus of research and application to replace racemic mixture (Pelosi et al., 2022). However, there was a significant time lag in current research on its environmental and ecological effects.

Current studies on the ecological effects of GLA in soil almost exclusively focused on its racemate. The main focus was on its impact on soil microbial community structure (Dennis et al., 2018; Zaller et al., 2018), enzyme activity (Deng et al., 2019; Zhang Z. Y. et al., 2019), and nutrient cycling (Reddy et al., 2011; Ghosh et al., 2017; Pattison et al., 2024). However, the uniqueness of L-GLA as the active moiety was generally overlooked--on the one hand, the high activity of L-GLA may enable it to exert specific effects on non-target organisms in soil even at a dose lower than the recommended dosage for the racemic configuration; on the other hand, the interaction between its molecular structure and soil components (such as organic matter, clay), and its non-targeted effect on functional microorganisms may differ fundamentally from that of racemate. These differences directly determined the actual ecological risks of L-GLA, which have not yet been systematically revealed.

The distinctive ecological risks of L-GLA are primarily manifested in two key dimensions, which was currently underexplored in existing racemic studies. ①Specificity of SOM binding and carbon pool impact: SOM is the core carrier of soil carbon cycle and the main carbon source for microorganisms (Witzgall et al., 2021). Early experiments and preliminary results of this study indicated that L-GLA may be more prone to disrupting the foundation of soil carbon stocks, thereby indirectly affecting microbial functions related to carbon cycling; ②Non-targeted inhibition of functional microorganisms: Deng et al. (2019) found that GLA inhibited the population growth of soil fungi under the concentration of 50 ~ 500 mg/kg, while promoted the population growth of soil bacteria and actinomycetes under low concentrations (50 ~ 100 mg/kg) and inhibited when exposed to high concentrations (250 ~ 500 mg/kg). Herbicides containing active ingredient glufosinate increased the total colony-forming units of soil microbes (Zaller et al., 2018). Albrecht and Kortekamp (2009) reported that GLA-based herbicides have antifungal properties. Furthermore, L-GLA showed the adverse effects on *Caenorhabditis elegans* (Zhao et al., 2023). Those non-targeted inhibition may disrupt the balance of the soil nitrogen cycle, and even further interfere with the carbon cycle metabolic function through the “carbon-nitrogen coupling” effect. However, this cross-cycle chain effect has not yet attracted researchers’ attention.

Given the potential applications and the lack of researches on ecological effects of L-GLA, elucidating its specific impacts on soil microbial community structure and carbon cycle metabolic functions has become a core requirement for assessing its environmental safety. Therefore, we combined metagenomics and pseudotargeted metabolomics based on Gas Chromatography–Mass Spectrometry (GC–MS) to systematically investigate the effect of different doses of L-GLA on soil microbial community composition, symbiotic networks, carbon cycle-related functional genes/enzyme activities, and metabolite profiles at different time points. The aim was to reveal the unique ecological effect mechanism of L-GLA that distinguished it from its racemic configuration, to fill the gap in the research on the non-target effects of this active isomer, and to provide a scientific basis for its safe application in agricultural production and ecological risk management.

## 2 Materials and methods

### 2.1 Experimental design and sample collection

Experiments were conducted in a greenhouse at Research Base of Guizhou Academy of Tobacco Science in Fuquan City (N26°32′28′′, E107°14′26′′), Guizhou Province, China. The soil used in this study was yellow-brown, and not sprayed with herbicides before the experiment. The physicochemical properties of 0–20 cm soil were shown in Supplementary Table S1. Three weeks before the experiment, weeds were pulled out of the test plot, the soil was turned over and the soil surface was leveled. A randomized block experimental design was used with a plot size of 6 m^2^ (3 m × 2 m), and the treatment plots were separated from each other by hand-dug trenches according to Wu et al. (2026) and made minor modification based on actual situation. The test soil was sprayed by water with automatic sprinkler facilities to keep adequately moistened. Tobacco plants (Yunyan 87) had been planted without herbicides application on April 12, 2023. The L-GLA (10% L-GLA soluble concentrate (SL); produced by Hailier Pesticides and Chemicals Group Co., Ltd.; Shandong, China) was applied on May 5, 2023, and it was sprayed onto the soil surface of the plots under three treatment conditions: (i) Control (CK), no L-GLA; (ii) L, the recommended dose (1×) (low) (600.00 g active ingredient (a.i.) hm^−2^); (iii) H, fivefold recommended dose (5×) (high) (3000.00 g a.i. hm^−2^). All tests were carried out in triplicate, cultured in greenhouse for 60 days based on previous studies (Rong et al., 2021; Zhang et al., 2022; Cao et al., 2023; Zhao et al., 2023). The temperature was 25 °C during the experiment in greenhouse, with addition of water during cultivation to keep adequately moistened.

Topsoil samples (0–20 cm) were collected using the five-point sampling method at day 30 and 60 from corresponding pots following L-GLA application. Samples were partitioned into two groups. One part underwent air-drying for assessing soil physicochemical properties. The other was stored at −80 °C for pesticide residue, metabolite analysis, and microbial examination.

### 2.2 Determination of L-glufosinate residue in the soil

L-glufosinate was extracted from the soil with an 0.1% formic acid water: methanol = 1:1 (v/v) mixed solvent. The L-glufosinate soil residue was determined by ultra-high-performance liquid chromatography coupled with tandem mass spectrometry (UHPLC–MS/MS) [ACQUTTY UPLC@BEH C_18_ (2.1 mm × 100 mm × 1.7 μm)] as described. The UHPLC–MS/MS chromatographic conditions included a mobile phase with chromatographic acetonitrile: ammonium acetate aqueous solution = 1:9 (v/v), column temperature of 35 °C, flow rate of 0.25 mL/min.

### 2.3 Soil properties analysis

Soil chemical parameters of Total nitrogen (TN), hydrolyzable nitrogen (HN), total phosphorus (TP), available phosphorus (AP), total potassium (TK), AK, SOM, and pH were determined in accordance with standard methods described by Bao (2000). TN, TP, and TK were determined utilizing Kjeldahl methodology combined with acid digestion. TN, AP, and AK were assessed by applying alkaline hydrolysis diffusion technique for nitrogen and the colorimetric method for phosphorus and potassium. Content of SOM was quantified through potassium dichromate oxidation. Soil pH was determined with a pH meter at a soil-to-water ratio of 1: 2.5.

### 2.4 Soil enzyme activity assay

The activities of soil sucrase (SC), amylase (AL), polyphenol oxidase (PPO), β-glucosidase (β-GC), and N-acetyl-β-D-glucosidase (NAG) were analyzed using commercial assay kits from Suzhou Comin Biotechnology Co., Ltd. (Jiangsu, China). The OD values of SC, AL, PPO, β-GC and NAG were determined at 510 nm, 540 nm, 430 nm, 400 nm and 400 nm, respectively.

### 2.5 Metagenomic analysis

#### 2.5.1 DNA extraction, Illumina sequencing

DNA samples from soil (*n* = 18, 0.5 g soil per sample) was extracted utilizing Magnetic Soil and Stool DNA Kit (TianGen Blotech Co., Ltd. China), concentration determination relied on NanoDrop 2000 UV–Vis spectrophotometer (Thermo Fisher Scientific, Wilmington, DE, USA). Resulting genomic DNA was randomly fragmented into 350 bp segments. Sequencing libraries were then prepared through end repair, the addition of sequencing adapters, and PCR amplification. The final libraries were sequenced using Illumina PE150 technology.

The metagenomic sequencing data was processed and analyzed in steps as follows. Firstly, sequencing results were conducted by Readfq^1^ to convert raw data into clean data (Karlsson et al., 2013). Next, the clean data was assembled into a metagenome using MEGAHIT software and Scaftigs without N were extracted by removing N junctions from the resulting scaffolds (Li et al., 2015). Abundance analysis and gene prediction were then performed with MetaGeneMark,^2^ which involved ORF prediction for Scaftigs (≥500 bp) from each sample (Oh et al., 2014). Finally, species and function were annotated by DIAMOND software.^3^ The involved aligning Unigene sequences can be referred from database like KEGG^4^ (Kanehisa et al., 2017) and Carbohydrate-Active Enzymes (CAZy, http://www.cazy.org/). Diversity of soil microbial communities was evaluated through alpha-diversity indices, computed by Mothur software (V.1.30.2).

#### 2.5.2 Network co-occurrence analysis

Symbiosis networks were analyzed to evaluate impact by L-GLA application on the interactions among soil microbial taxa at phylum and genus level. Analysis was based on significant Pearson correlation (*r* > 0.8, *p* < 0.05). Network analysis and visualization were conducted utilizing Gephi 0.10 (Csardi and Nepusz, 2006). Topological parameters, including mean path length, edges and nodes, average degree, diameter of network, were computed on Gephi platform.

### 2.6 Soil environmental pseudotargeted metabolomics analysis

Extraction and derivatization for soil metabolites and analysis on GC–MS environmental pseudotargeted metabolomics were performed by previous methods with minor modification (Cai et al., 2023). 0.5 g soil sample was processed in a 10 mL centrifuge tube. It underwent two times of vortex-assisted extraction with a methanol–water mixture (3,2, v/v), followed by a final extraction with water. The extract was freeze-dried overnight at −10 °C. For derivatization, the sample was treated with 20 μL of methoxyamine hydrochloride (25 mg/mL) for oximation, followed by 100 μL of N, O-bis(trimethylsilyl)trifluoroacetamide (containing 2% trimethylchlorosilane) for silylation. The derivatized products were analyzed using GC–MS. The extraction and derivatization procedures, as well as the GC conditions draw on the approach of Cai et al. (2023). Metabolites were identified by comparison with the NIST14 and Wiley08 databases, retention indices, and standard compounds, with purity evaluated using AMDIS software.

### 2.7 Data analysis

The residues of L-glufosinate in the soil were fit to a nonlinear regression model in Origin 2024b (OriginLab Corp., Northampton, MA, USA) software. The formula is as follows:

$$C_{t}=C_{0}\times e^{-\mathrm{kt}}$$

Where C_t_ is the L-glufosinate concentration at time t, C_0_ represents the L-glufosinate concentration at the initial time, and k represents the first-order rate constant. The half-life (*t_1/2_*) represents the time required for the L-glufosinate to degrade to 50% of its initial concentration and is calculated based on the formula *t_1/2_* = (ln2)/k.

One-way ANOVA in SPSS 25.0 was used to compared soil physicochemical properties, enzymatic activity, gene abundance related to carbon cycles, and microbial alpha-diversity indices between different treatments. All statistic analysis were carried out with three replicates. Statistical significance: *p* < 0.05. Constrained principal coordinate analysis (PCoA) were conducted by using Bray-Curtis distances to evaluate the difference within structure of microbial community at the genus level. Bar and stacked graph, and heatmaps were drawn via Origin 2024b (OriginLab Corp., Northampton, MA, USA). Characteristic species, functional metabolism and carbohydrate active enzymes genes were analyzed by STAMP v2.1.3. Principal components analysis (PCA) and volcano plot were conducted via Metaboanalyst 6.0.^5^ Fold change (FC) value >2.0 and false discovery rate (FDR) *p*-value <0.05 was applied to evaluate differential metabolite. Statistical analysis of characteristic metabolites and correlation between CK and treatments were performed using the Biozeron Cloud Platform (Wang et al., 2024). The characteristic metabolites were collected to analyse the biological pathways.

## 3 Results

### 3.1 Dissipation behaviors of L-glufosinate in soil environment

The residue of L-glufosinate in soil showed that L-glufosinate was detected in the treated groups, with its concentration gradually decreasing as the exposure time increased (Supplementary Figure S1). Specifically, the concentration of L-glufosinate in the recommended dose treatment (1×) decreased by 88.78%, while it decreased by 76.23% in the fivefold recommended dose treatment (5×). The half-life revealed that there was a significant difference in the recommended dose treatment and fivefold recommended dose treatment. The DT_50_ of L-glufosinate in the recommended dose treatment was 18.8 days, while it was 29.7 days in fivefold recommended dose treatment, indicating that the high-dose of L-glufosinate treatment was more persistent in the soil.

### 3.2 Effects of L-GLA on soil physicochemical properties

Pesticides that remain in the soil for a long time often affect the physical and chemical properties of the soil. Therefore, we analyzed the effect of L-GLA stress on soil physicochemical properties after 30 and 60 days of application (Supplementary Table S1). The results showed that after 30 days of L-GLA stress, both L and H treatment significantly reduced the content of SOM (*p* < 0.05), with the H treatment having a more significant effect on SOM content reduction. Although the SOM content in the treated groups had recovered at 60 days after application, it was still lower than the control group. The content of AK significantly decreased at both dosed treatments and TK decreased in L treatment. At the same time, the nutrient concentrations such as TN, AN, TP, and AP did not reach a significant level whether treated with L-GLA for 30 or 60 days. These results indicated that L-GLA exposure significantly affected the carbon cycle, while its impact on other major soil nutrients was relatively minor.

### 3.3 Effects of L-GLA on soil microorganisms

#### 3.3.1 Changes in soil microbial diversity and community composition due to L-GLA application

**Figure 1:**
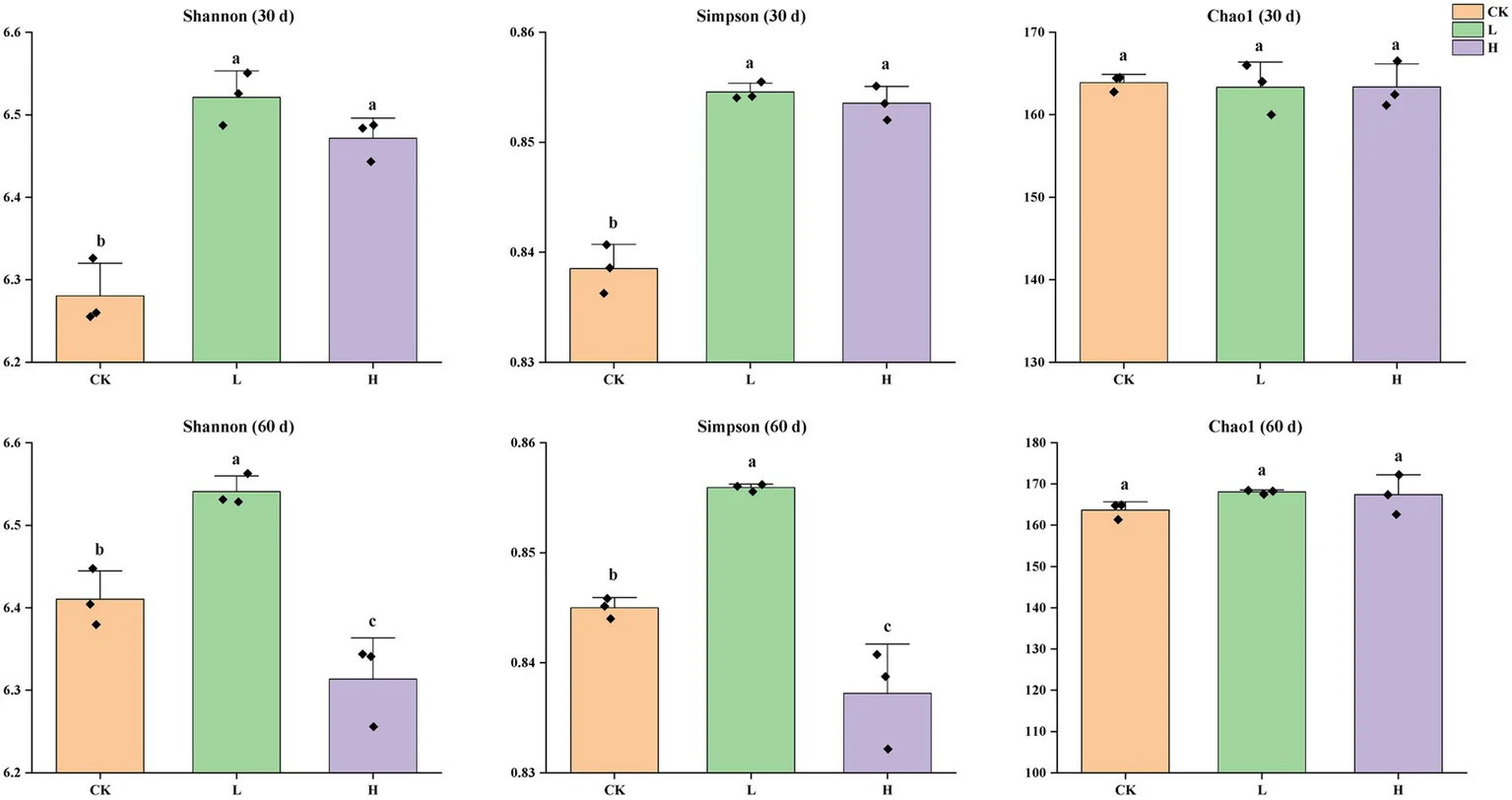
Comparison of alpha diversity based on Shannon, Simpson and Chao 1 indexes between the control and two-concentration treatments. CK: Without L-glufosinate-ammonium addition; L: Application at 600 g a.i. hm^−2^; H: Application at 3000 g a.i. hm^−2^. Lowercase letters (a, b, c) indicate significant differences among different treatments for a given time point using one-way ANOVA (*p* < 0.05).

**Figure 2:**
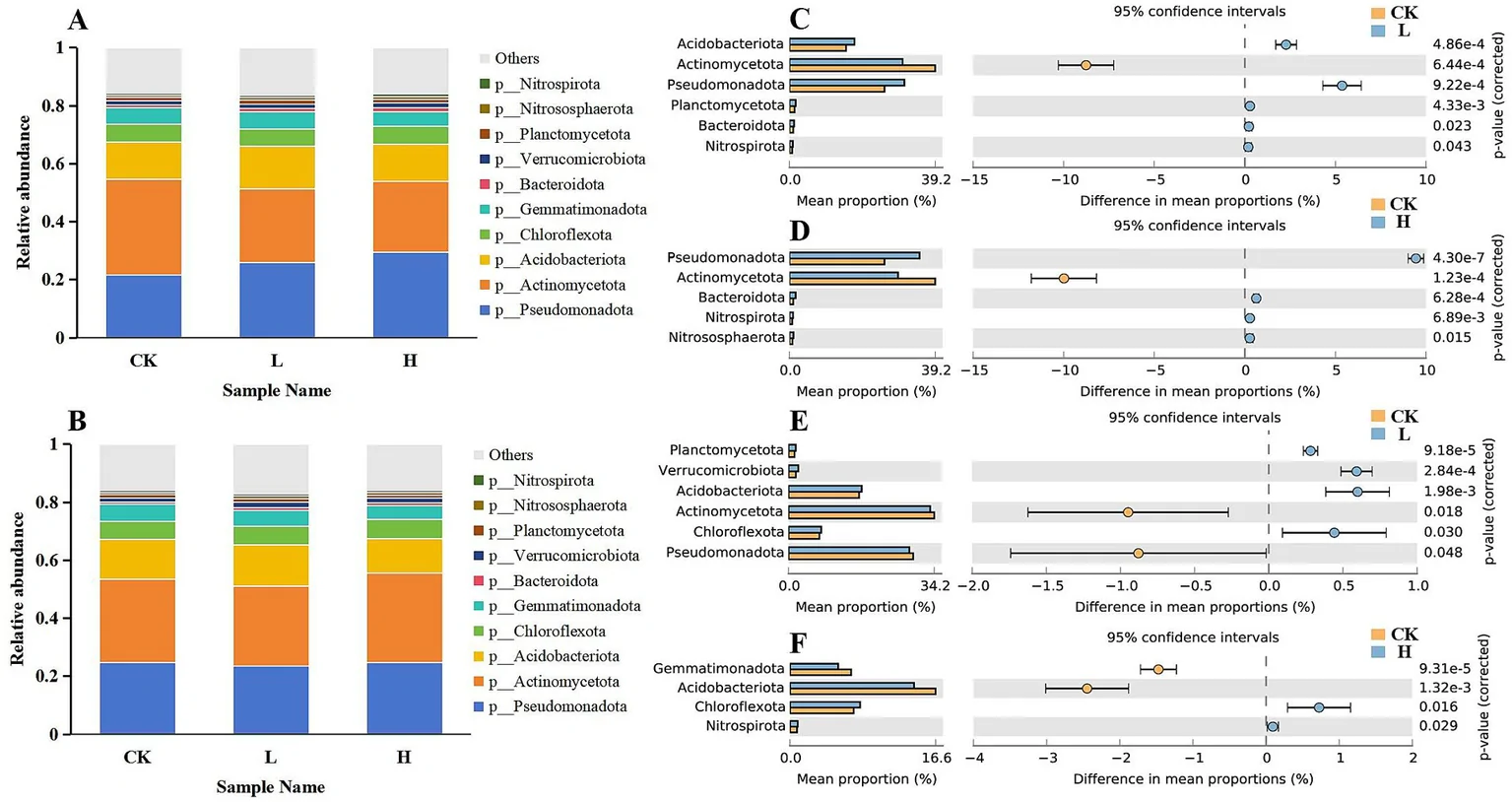
Relative abundances histograms of microbial community (top 10) at the phylum level and differential phyla between CK and treatments. **(A,B)** The community composition at 30 and 60-post application, respectively; **(C,E)** Differential phyla between CK and L on day 30 and 60, respectively; **(D,F)** Differential phyla between CK and H on day 30 and 60, respectively. CK: Without L-glufosinate-ammonium addition; L: Application at 600 g a.i. hm^−2^; H: Application at 3000 g a.i. hm^−2^.

There is a close relationship between the soil physicochemical properties and soil microbes. Microbes can influence the soil physicochemical properties by participating in nutrient cycling and organic matter transformation. Therefore, we measured the impact of L-GLA residues on soil microbes. The index of Shannon, Simpson and Chao 1 were used to assess the impact of L-GLA exposure on the alpha diversity of soil microbes (Figure 1). The results showed that after 30 days of exposure, both the L and H treatments experienced significant increase in Shannon and Simpson indices, indicating an initial increase in microbial richness and evenness. However, after 60 days of exposure, the L treatment maintained a high level of Shannon and Simpson, while the H treatment showed a significant decrease. In addition, the PCoA analysis revealed that the microbial *β*-diversity changed under different doses and exposure times (Supplementary Figure S2). Furthermore, the change in the community composition were analyzed (Figure 2A,B; Supplementary Figure S3). After 30 days of L-GLA exposure, the result revealed that the abundance of Actinomycetota decreased significantly, and the abundance of Acidobacteriota, Pseudomonadota, Planctomycetota, Bacteroidota, and Nitrospirota increased notably in L treatment at the phylum level (Figure 2C). The H treatment resulted in a significant decrease in the abundance of Actinomycetota and a significant increase in the abundance of Pseudomonadota, Bacteroidota, Nitrospirota, and Nitrososphaerota (Figure 2D). At the genus level, the abundance of *Intrasporangium*, *Marmoricola*, *Conexibacter*, *Aeromicrobium*, *Nocardioides*, *Solirubrobacter*, *Streptomyces*, *Kribbella* and *Gaiella* decreased markedly, and the abundance of *Ramlibacter*, *Terrabacter*, *Acidobacterium*, *Steroidobacter*, *Novosphingobium*, *Mesorhizobium*, *Luteitalea*, *Massilia*, *Hyphomicrobium*, *Lysobacter*, *Flavihumibacter*, *Pseudorhodoferax*, *Sphingosinicella*, *Humisphaera*, *Arthrobacter*, *Pseudarthrobacter*, *Sphingomonas*, *Nitrospira*, *Nitrososphaera* and *Agromyces* enhanced significantly in the L treatment (Supplementary Figure S4A). The changes in the H treatment are more complex, specifically manifested as a noticeable decrease in the abundance of *Intrasporangium*, *Marmoricola*, *Solirubrobacter*, *Conexibacter*, *Streptomyces*, *Gaiella*, *Desertimonas*, *Roseisolibacter*, *Bradyrhizobium*, *Hyphomicrobium*, *Agromyces*, *Gemmatirosa*, and a markedly increase in the abundance of *Steroidobacter*, *Luteitalea*, *Luteimonas*, *Ramlibacter*, *Nitrososphaera*, *Massilia*, *Terrabacter*, *Pseudorhodoferax*, *Lysobacter*, *Sphingopyxis*, *Serratia*, *Pseudomonas*, *Advenella*, *Mesorhizobium*, Var*iovorax*, *Nocardioides*, *Flavihumibacter*, *Phenylobacterium*, *Cellvibrio*, *Nitrospira*, *Brevundimonas*, *Acidobacterium*, *Microbacterium*, *Usitatibacter*, *Olivibacter* and *Novosphingobium* (Supplementary Figure S4B). After 60 days of exposure, at the phylum level, the L treatment resulted in a significant decrease in the abundance of Actinomycetota and Pseudomonadota, and a significant increase in the abundance of Acidobacteriota, Chloroflexota, Planctomycetota and Verrucomicrobiota (Figure 2E). Exposure to the H treatment resulted in a significant decrease in the abundance of Gemmatimonadota and Acidobacteriota, and a significant increase in the abundance of Chloroflexota and Nitrospirota (Figure 2F). At the genus level, the L treatment resulted in a significant decrease in the abundance of *Gemmatirosa*, *Sphingosinicella*, *Pseudarthrobacter*, *Novosphingobium*, *Sphingomonas*, *Roseisolibacter*, *Intrasporangium*, and *Arthrobacter*, while the abundance of *Pseudomonas*, *Acidobacterium*, *Humisphaera*, *Steroidobacter*, *Marmoricola*, *Streptomyces*, *Mesorhizobium*, *Kribbella*, *Massilia*, *Pseudorhodoferax*, *Sphingobacterium*, and *Nitrososphaera* increased significantly (Supplementary Figure S4D). The H treatment dramatically decreased the abundance of *Intrasporangium*, *Agromyces*, *Gemmatirosa*, *Bradyrhizobium*, *Arthrobacter*, *Roseisolibacter*, *Solirubrobacter*, *Gaiella*, *Hyphomicrobium*, *Desertimonas*, *Reyranella*, *Sphingosinicella*, *Novosphingobium*, *Humisphaera*, *Acidobacterium*, *Pseudarthrobacter*, *Luteitalea*, *Usitatibacter* and markedly improved the abundance of *Terrabacter*, *Ramlibacter*, *Lysobacter*, *Aeromicrobium*, *Sphingobacterium*, *Microbacterium*, *Mesorhizobium*, *Nitrososphaera*, *Pseudomonas*, *Streptomyces*, *Conexibacter*, *Kribbella*, *Marmoricola*, *Nocardioides*, *Phenylobacterium* and *Massilia* (Supplementary Figure S4C). These results indicated that L-GLA exposure significantly altered the microbial community diversity and compositions, and exhibited significant dose- and time-dependent effects. High dose exposure had a more intense and detrimental impact on the microbial community structure.

#### 3.3.2 Dose-dependent regulation of soil microbial community interactions by L-GLA

**Figure 3:**
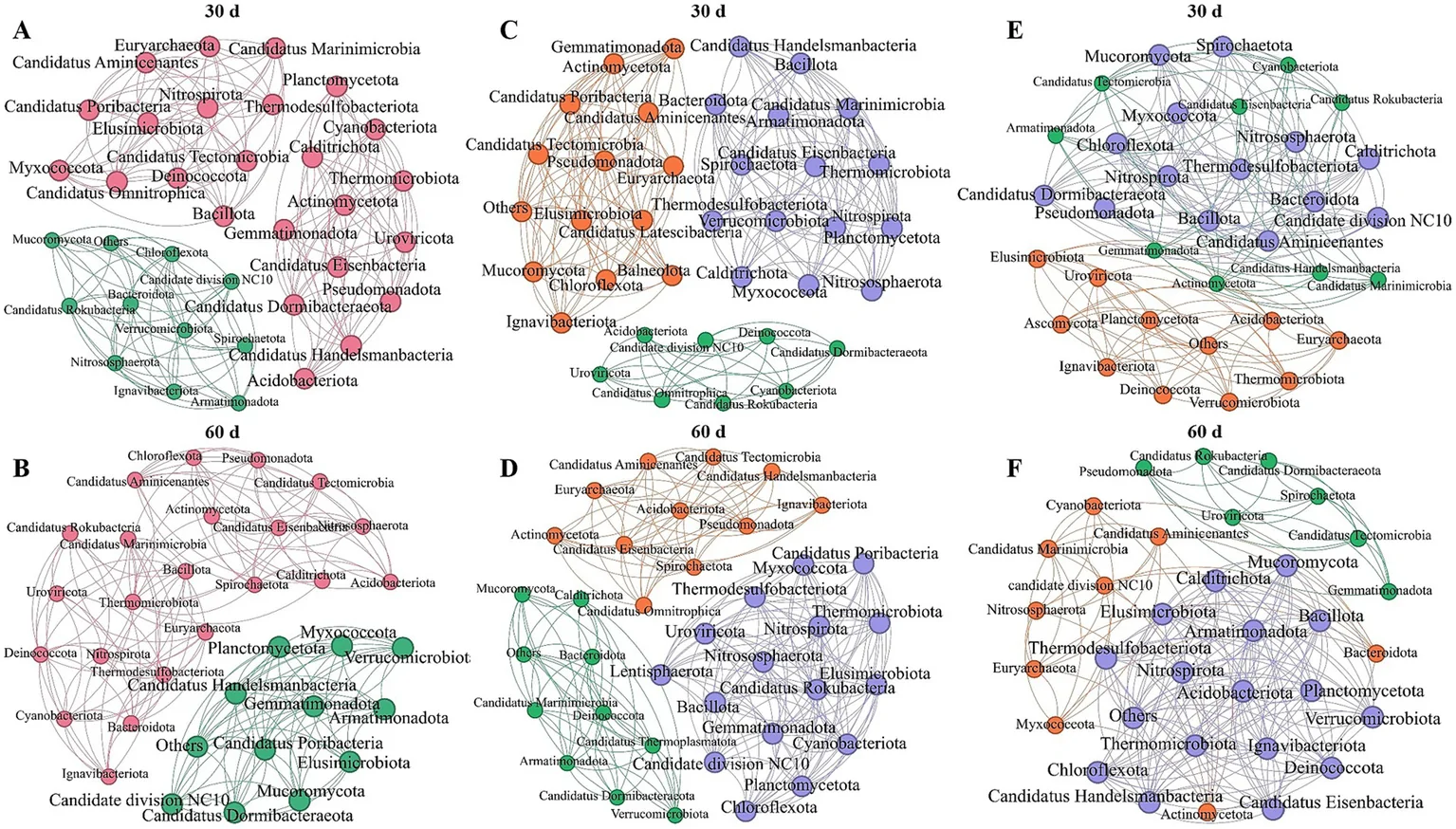
Network co-occurrence analysis of microbial communities of different treatments (at phylum level). A connection stands for a strong (Spearman’s |*r*| > 0.7) and significant (*p* < 0.05) correlation for the control **(A,B)** CK and L-glufosinate-ammonium treatments **(C,D)** L, 600 g a.i. hm^−2^; **(E,F)** L, 3,000 g a.i. hm^−2^. The size of each node is proportional to its relative abundance.

Subsequently, a soil bacterial network was constructed to reveal the potential impact of L-GLA on the co-occurrence patterns of bacterial communities. As shown in the Figure 3 and Supplementary Table S2, after 30 days of application, the number of nodes and edges, and the average degree in the L treatment (37, 224, and 12.108) were all higher than those in the control group (35, 187, and 10.686). At the same time, the number of module was slightly lower than that of the control (0.6 vs. 0.664), indicating that the L treatment increased the connection strength and complexity of the network. For the H treatment, although there was no significant difference in the number of nodes, edges, and the average degree compared to the control, the number of module was much lower than that of the control (0.62 vs. 0.664), and it also increased the network complexity. After 60 days of exposure, the L and H treatments continued to exhibit higher connection strength and network complexity. Specifically, the number of nodes and edges, and the average degree (37, 220, and 11.892) in the L treatment were significantly higher than those in the control group (34, 176, and 10.353), while the number of module was slightly lower than that of the control group (0.598 vs. 0.664). For the H treatment, the number of module was still significantly lower than that of the control group (0.457 vs. 0.664). Moreover, the number of edges and the average degree (193 and 11.697) of the H treatment were also higher than those of the control group. These results indicated that L-GLA significantly altered the interactions among soil bacterial communities and exhibited a significant dose-dependent effect.

#### 3.3.3 Regulation of soil microbial metabolic functions

The impact of L-GLA stress on soil microbial metabolic functions was studied based on the KEGG database (Supplementary Figure S5). After 30 days of exposure, the L treatment showed a significant change in 12 metabolic pathways compared with the control group. These metabolic pathways included two-component system, starch and sucrose metabolism, cysteine and methionine metabolism, fatty acid degradation, bacterial secretion system, glyoxylate and dicarboxylate metabolism, valine, leucine and isoleucine degradation, homologous recombination, arginine and proline metabolism, arginine biosynthesis, carbon fixation pathways in prokaryotes and glycolysis/gluconeogenesis. The group treated with a fivefold recommended dose induced significant change in 17 metabolic pathways, including glyoxylate and dicarboxylate metabolism, cysteine and methionine metabolism, bacterial secretion system, two-component system, starch and sucrose metabolism, aminoacyl-tRNA biosynthesis, pentose phosphate pathway, arginine biosynthesis, carbon fixation pathways in prokaryotes, nitrogen metabolism, alanine, aspartate and glutamate metabolism, butanoate metabolism, glycolysis/gluconeogenesis, pyruvate metabolism, ABC transporters, purine metabolism and oxidative phosphorylation. After 60 days of exposure, although the impact on microbial functional metabolism was alleviated, there was still difference in three metabolic pathways, namely nitrogen metabolism, homologous recombination and two-component system, between the L treatment and the control. Furthermore, there were 11 metabolic pathways that showed significant differences between the control group and the H treatment. These pathways include fatty acid degradation, valine, leucine and isoleucine degradation, porphyrin metabolism, quorum sensing, arginine and proline metabolism, pentose phosphate pathway, cysteine and methionine metabolism, propanoate metabolism, ABC transporters, tryptophan metabolism and homologous recombination. These results indicated that L-GLA significantly altered the microbial functional metabolism and exhibited significant dose- and time-dependent effects.

#### 3.3.4 Effect of L-GLA on soil microbial carbohydrate-active enzyme genes and its dose response

**Figure 4:**
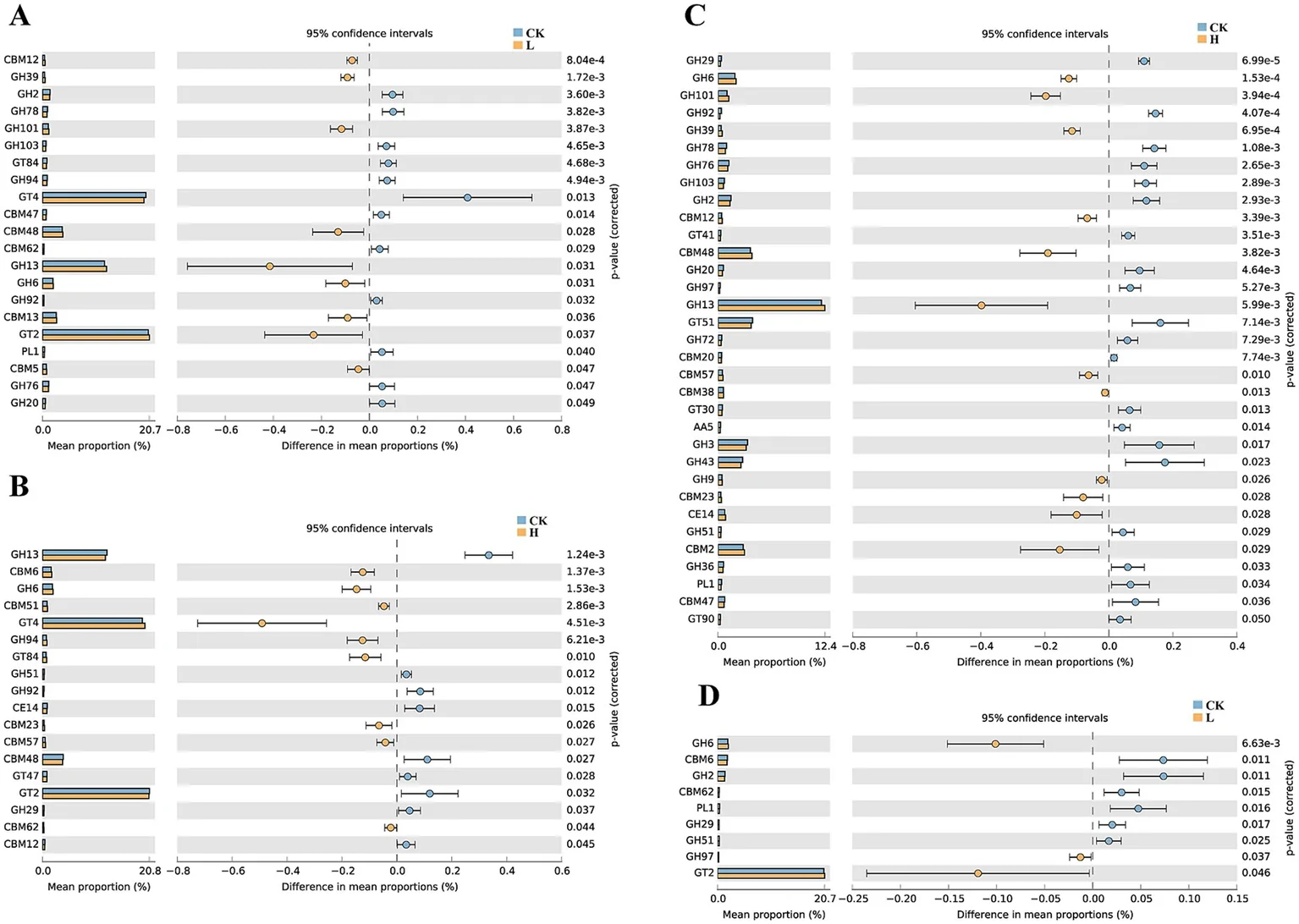
Characteristic microbial carbohydrate active enzymes genes based on CAZyme database at level 2. **(A,D)** Characteristic CAZyme genes between CK and L on day 30 and 60, respectively; **(C,B)** Characteristic CAZyme genes between CK and H on day 30 and 60, respectively. CK: Without L-glufosinate-ammonium addition; L: Application at 600 g a.i. hm^−2^; H: Application at 3,000 g a.i. hm^−2^.

Further analysis was conducted to uncover the effect of L-GLA on carbohydrate active enzymes (CAZymes) genes (Figure 4). After 30 days of application, the L treatment resulted in significant change in the abundance of 21 CAZymes genes, specifically including CBM12, CBM47, CBM48, CBM62, CBM13, CBM5, GH39, GH2, GH78, GH101, GH103, GH94, GH13, GH6, GH92, GH76, GH20, GT84, GT4, GT2 and PL1. And the H treatment affected more types of CAZymes genes, including GH29, GH6, GH101, GH92, GH39, GH78, GH76, GH103, GH2, GH20, GH97, GH13, GH72, GH3, GH43, GH9, GH51, GH36, CBM12, GT51, GT30, GT90, CBM20, CBM57, CBM38, CBM2, CBM47, AA50, CE14 and PL1. After 60 days of exposure, the effect of L-GLA stress on CAZymes genes remained significantly. The L treatment induced in significant changes in the abundance of nine CAZymes genes, such as GH6, CBM6, GH2, CBM62, PL1, GH29, GH51, GH97 and GT2. The H treatment affected 18 types of CAZymes genes, including GH13, CBM6, GH6, CBM51, GT4, GH94, GT84, GH51, GH92, CE14, CBM23, CBM57, CBM48, GT47, GT2, GH29, CBM62 and CBM12. These results indicated that L-GLA exerted a significant effect on the CAZymes genes at both time points, and the impact increased with the increase in dosage.

### 3.4 Effects of L-GLA on the enzymes activities related to soil carbon cycling

The activity of enzymes related to the soil carbon cycle were further measured (Supplementary Figure S6). The results revealed that the L treatment significantly influenced the activity of N-acetyl-β-D-glucosidase, sucrase, and β-glucosidase after 30 days of application. While the H treatment had a more severe impact on the activity of these enzymes, and also significantly altered the activity of soil amylase. After 60 days of application, the L treatment caused changes in the activity of soil polyphenol oxidase, soil amylase and N-acetyl-β-D-glucosidase. Meanwhile, treatment with fivefold recommended dose of L-GLA further led to significant change in soil amylase and β-glucosidase activity. These results indicated that the application of L-GLA had a significant effect on the activity of soil enzyme.

### 3.5 Effects of L-GLA on soil metabolites

#### 3.5.1 Changes in metabolite spectrum

The change in composition and content of soil metabolites can reveal the direct response of soil microorganism to soil nutrient. The influence of L-GLA on the soil metabolic profile was explored by using soil environmental pseudotargeted metabolomics. A total of 187 metabolites were identified from the soil samples. PCA analysis revealed that both L and H treatments showed a distinct separation trend from the control group at both 30 and 60 days after application, suggesting that the soil metabolic profile had undergone significant change after L-GLA exposure (Supplementary Figure S7). Then, based on the criteria of FC > 2.0 and FDR *p* < 0.05, characteristic metabolites that showed significant change under L-GLA exposure were screened. The results showed that the L treatment led to significant change in 43 metabolites at 30 days after application (Supplementary Figure S8A). Among them, the abundance of maltitol, ribonic acid, spermidine, maleic acid, 1-ethoxy-2-propanol, terephthalic acid, decanoic acid and gamma-aminobutyric acid increased significantly, while the abundance of methyl galactoside, phthalic acid, isobutyl undecyl ester, xylitol, dodecanoic acid, tetradecenoic acid, 3-Hydroxy-2-methylbutyric acid, 2,5-Dihydroxybenzoic acid, stearic acid, abietic acid, sorbitol, methyl-α-D-glucopyranoside, pipecolinic acid, dehydroabietic acid, galactinol, putrescine, uracil, leucine, myristic acid, alanine, valine, 9-Hexadecenoic acid isomer, glutamic acid, melibiose 1, serine, ethanolamine, pimaric acid, hexadecanoic acid, cadaverine, mannitol, 1-octadecanol, 1-hexadecanol, isoamylamine, D-galactose, myo-Inositol, 3-hydracrylic acid decreased significantly. After 30 days of treatment with fivefold recommended dose of L-GLA, significant change was observed in 71 metabolites (Supplementary Figure S8B). Among them, the abundance of pinitol, ribonic acid, malic acid, turanose, serine, guanine, maleic acid, benzeneacetic acid, oxalic acid, abietic acid, pipecolinic acid, methyl-α-D-glucopyranoside, proline, syringic acid, glycerol-3-phosphate, dehydroabietic acid, phosphate, shikimic acid, phthalic acid, isobutyl undecyl ester, pimaric acid, rhamnose, myo-Inositol, 2,3-butanediol, glyoxylic acid, 3-hydroxybutyric acid, p-coumaric acid, 4-hydroxyphenylacetic acid, monomethyl phosphate and arabinitol increased obviously, while the abundance of 1-monopalmitin, vanillic acid, mannitol, 2,4-dihydroxybenzoic acid, ferulic acid, erythritol, cadaverine, 3-hydracrylic acid, glycerophosphoglycerol, diethylene glycol, uracil, 2,2-dimethyl-3-pentanol, hexadecanoic acid, phosphoethanolamine, triethylene glycol, 1-pentadecanol, gamma-aminobutyric acid, 1,2-benzenedicarboxylic acid, bis(2-methylpropyl) ester, glycerol 1-monostearate, galactinol, urea 2, Isoleucine, sorbitol, ethanolamine, glucose 1, putrescine, cinnamic acid, vanillin, leucine, glycine, valine, 1-octadecanol, glyceryl-glycoside, mannonic acid *γ*-lactone, 1-hexadecanol, alanine, threonine, isoamylamine, disaccharide, 2,5-dihydroxybenzoic acid, tyrosine, D-galactose decreased dramatically. After 60 days of exposure, the composition and content of soil metabolites recovered to some extent, but there were still 25/44 metabolites that showed significant differences between the L treatment/H treatment and the control group (Supplementary Figures S8C,D). These data indicated that L-GLA exposure significantly altered the metabolites composition, and this effect exhibited a certain dose-dependent and temporal dynamics.

#### 3.5.2 Differential pathway enrichment analysis

**Figure 5:**
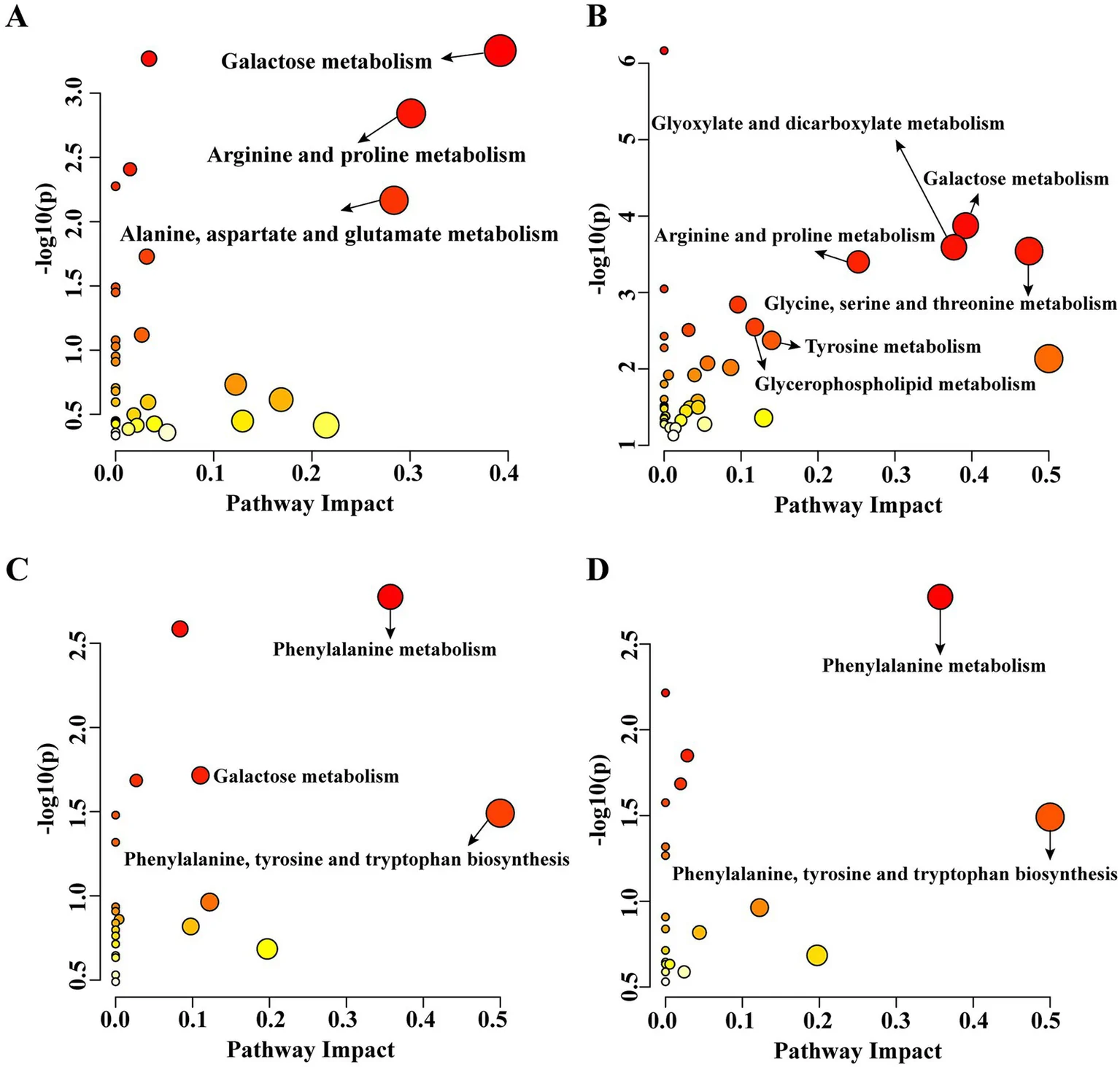
Bubble diagram of metabolic pathways that were significantly different between CK and treatments (significant pathways with pathways impact value >0.1 and *p* < 0.05 were marked). **(A,C)** Significant pathways between CK and L at 30 and 60 post-application, respectively; **(B,D)** Significant pathways between CK and H on day 30 and 60, respectively. CK: Without L-glufosinate-ammonium addition; L: Application at 600 g a.i. hm^−2^; H: Application at 3,000 g a.i. hm^−2^.

Analysis delved into the enrichment pathways using the KEGG compounds of the differential metabolites. Screening with pathways impact value >0.1 and *p* < 0.05 as thresholds marked 10 key metabolic pathways in a bubble chart (Figure 5). Pathway analysis revealed that significant changes (pathways impact >0.1 and *p* < 0.05) have occurred in galactose metabolism, glyoxylate and dicarboxylate metabolism, phenylalanine, tyrosine and tryptophan biosynthesis, phenylalanine metabolism, glycine, serine and threonine metabolism, alanine, aspartate and glutamate metabolism, arginine and proline metabolism, tyrosine metabolism, glutathione metabolism and glycerophospholipid metabolism. These significant affected pathways mainly contained metabolism, including carbohydrate metabolism, amino acid metabolism, lipid metabolism and metabolism of other amino acids. It can inferred that the L-glufosinate interfered with the basic metabolism, such as carbohydrate, amino acid and glycerolipid metabolism.

### 3.6 Correlation between differential microorganisms and characteristic metabolites

**Figure 6:**
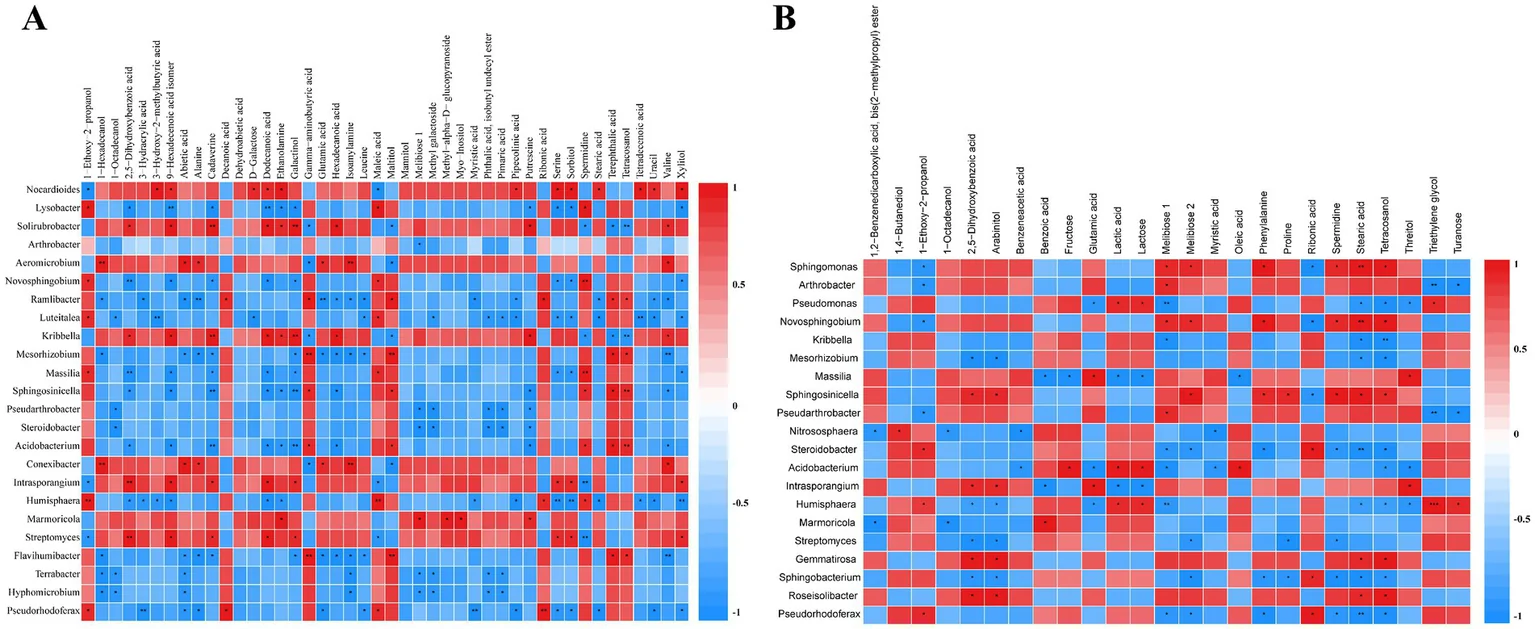
Correlation of the differential genera, in terms of relative abundance, with the differential metabolites between CK and L at 30 d **(A)** and 60 d **(B)**, respectively. The vertical and horizontal axis represents differential genera and differential metabolites, respectively. Red and blue indicate positive and negative correlation, respectively. * and ** represent significance (*p* < 0.05) and high significance (*p* < 0.01).

**Figure 7:**
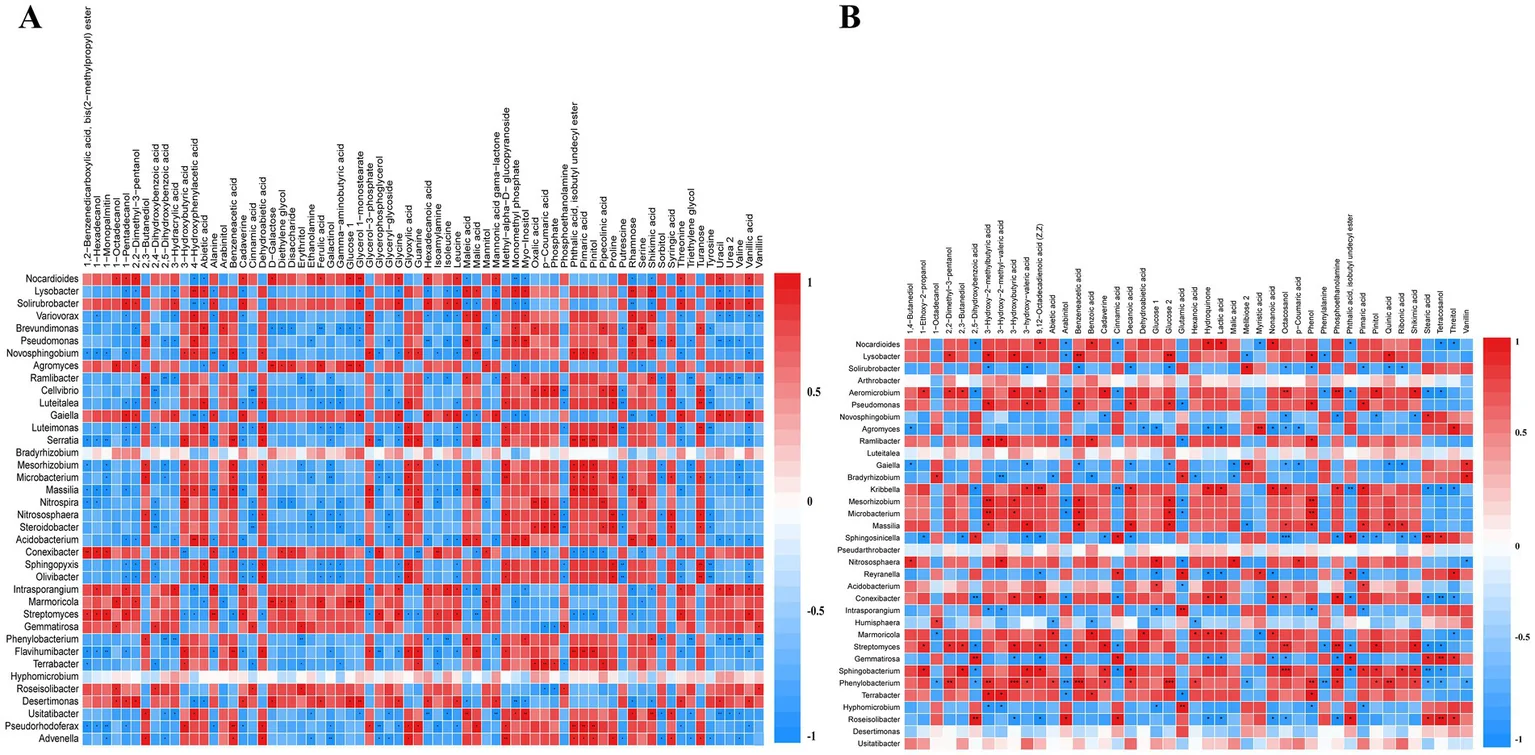
Correlation of the differential genera, in terms of relative abundance, with the differential metabolites between CK and H at 30 d **(A)** and 60 d **(B)**, respectively. The vertical and horizontal axis represents differential genera and differential metabolites, respectively. Red and blue indicate positive and negative correlation, respectively. * and ** represent significance (*p* < 0.05) and high significance (*p* < 0.01).

The correlation between differential microbes at the genus level and characteristic metabolites above were determined based on the abundance. Significant (*p* < 0.05) and highly significant (*p* < 0.01) correlations were observed linking the differential genera with differential metabolites (Figure 6). Generally, there were more negative correlations (blue) than positive correlations (red) between metabolites and microbes in the heatmaps. For the differential microbes, seven genera, including *Novosphingobium*, *Mesorhizobium*, *Massilia*, *Acidobacterium*, *Intrasporangium*, *Marmoricola* and *Streptomyces*, were simultaneously present at 30 and 60-post application, respectively. *Novosphingobium*, *Mesorhizobium*, *Massilia* and *Acidobacterium*, were negatively correlated with most metabolites on day 30. For example, *Novosphingobium*, *Acidobacterium*, and *Massilia* was significantly negatively correlated with the abundance of 2,5-dihydroxybenzoic acid, 9-hexadecenoic acid, cadaverine, dodecanoic acid and galactinol. While *Intrasporangium*, *Marmoricola* and *Streptomyces* showed the opposite result. These three genera were positively correlated with the abundance of 1-hexadecanol, 1-octadecanol, 2,5-dihydroxybenzoic acid and 3-hydracrylic acid. For the differential metabolites, 1-Octadecanol and 2,5-Dihydroxybenzoic acid with decreased abundances were simultaneously present in two groups at 30 and 60-post application, respectively. These two metabolites were negatively correlated with the increased abundance of *Novosphingobium*, *Mesorhizobium*, *Massilia*, and *Acidobacterium*, and were positively correlated with the decreased abundance of *Intrasporangium*, *Marmoricola* and *Streptomyces.* Therefore, it may be inferred that the microbes with increased abundance favored their growth with 1-Octadecanol and 2,5-Dihydroxybenzoic acid consumption. The second frequency of occurrence of differential metabolites were 1-Ethoxy-2-propanol, glutamic acid, myristic acid, ribonic acid, stearic acid, tetracosanol, abietic acid, cadaverine, phthalic acid, isobutyl undecyl ester and pimaric acid (see Figure 7).

## 4 Discussion

This study explored the impact of L-GLA (rather than the racemic configuration studied previously) on the yellow-brown soil microenvironment by integrating metagenomics with GC–MS soil environmental pseudotargeted metabolomics. We found that the degradation of L-GLA was dose-dependent. The application of L-GLA significantly perturbed SOM and AK (no significant impact on other nutrients), reconstructed microbial communities and symbiotic networks, interfered with carbon cycle functions, and altered the metabolite profile. Overall, previous study revealed the unique ecological effects of L-GLA as an active isomer, filling the gap in the research on the non-target effects of this isomer (Thiour-Mauprivez et al., 2019).

Due to the dual effects of “enzyme saturation - sensitive bacteria inhibition” (Tudararo-Aherobo and Ataikiru, 2020), high-dose of L-GLA degraded more slowly—compared to the L treatment, the activity of catalase decreased by 4.03%, and the abundance of sensitive degrading bacteria *Solirubrobacter* decreased by 29.83% at 30 d in the H treatment; the observed changes in soil physicochemical properties were consistent with a strategy of “carbon reservoir targeting” (Zhang et al., 2022); by day 30, the H treatment (8.7%) had exhibited a significantly decrease in SOM compared to the L treatment (4.6%), the mechanism involved the strong binding of L-GLA to the carboxyl groups of SOM (direct inhibition of decomposition) and the accelerated consumption of SOM by the enrichment of Bacteroidota (indirect driving). This contrasted with the effect of the racemate “broad-spectrum influence on nutrient cycling”(Kopčáková et al., 2016), and highlighted the necessity of exploring the sole effect of L-GLA.

Both treatments improved the microbial *α*-diversity at 30 days, and it showed a significant decrease only in the H treatment at 60 days. In terms of community composition, the H treatment was enriched with Pseudomonadota and *Lysobacter*, suggesting that the application of L-GLA may recruit the probiotics (Zhang L. et al., 2019; Zhang Z. Y. et al., 2019).

The application of L-GLA led to “increased complexity but decreased modularity” in the symbiotic network. By 30 days post-treatment, the L treatment exhibited a significantly higher network complexity, as evidenced by an increased number of nodes and edges compared to the control group, while a significantly lower modularity was observed in the H treatment relative to the control. The above characteristics were maintained at the 60-day time point. These models differed from dicamba (reducing the frequency of co-occurrence) (Shao et al., 2025) and clomazone (only increasing the network connectivity) (Rong et al., 2021). Decreased modularity within the symbiotic network served as an indicator for a convergence of functional module boundaries and an attenuation of the functional redundancy in the community (Banerjee et al., 2016; Jiao et al., 2019). The H treatment exhibited a 42% reduction in its keystone species composition. Consequently, the community exhibited a compromised resilience against subsequent environmental perturbations (Cao et al., 2023). The “network topological parameter” in this research offered a new indicator for the ecological risk assessment of herbicides.

The disturbance of the carbon cycle induced by L-GLA was strongly dose-dependent, with higher concentrations leading to more sustained effects. At the 30-day mark, the H treatment exerted an impact on 33 CAZymes genes (21 genes affected in the L treatment), the 41.3% down-regulation of GH39 directly resulted in a 35% reduction in its enzymatic activity; at the enzymatic level, the H treatment elicited a sustained suppression of both amylase and β-glucosidase activities, which remained statistically significant even at day 60; at the metabolic pathway level, the H treatment perturbed 17 carbon-associated pathways (e.g., the glyoxylate cycle, carbon fixation), thereby establishing a causal chain of “CAZymes genes - carbon cycle enzymes - metabolic pathways” (Dennis et al., 2018; Cao et al., 2023). Moreover, this intervention exhibited stronger targeting specificity toward the carbon cycle compared to the broad-spectrum effects exerted by the racemic mixture. These findings offered a more sophisticated mechanistic perspective on herbicide-driven disruptions to soil carbon cycling (Zhang et al., 2021; Zhang et al., 2022).

Differential metabolites, including 1-octadecanol and 2,5-dihydroxybenzoic acid, were identified as potential biomarkers for L-GLA stress based on their significant associations with specific microorganisms. In a word, this work systematically studied the microecological effects of L-GLA on soil, and constructed a multi-scale analysis framework for herbicide “environmental behavior--functional response--metabolic regulation” in yellow brown soil (Cai et al., 2023). To a certain extent, present work had its practical implications for L-GLA application and environmental risk assessment (Cao et al., 2023). It is worth pointing out that the yellow-brown soil was the main soil type in Guizhou, L-GLA is a commonly used herbicide in tobacco production and the DT_50_ of L-glufosinate in the recommended dose treatment was 18.8 days, so we choose the greenhouse and yellow-brown soil. In the future work, more comprehensive understanding and refinement of these findings will entail future studies that employ field positioning to expand the generalizability to real-world agricultural setting, encompass multiple soil types for broader applicability, adopt an integrated “crop-soil-microbiome” systemic approach, complemented by long-term monitoring, framework, and include long-term observation (Ren et al., 2024; Pattison et al., 2024; Wen et al., 2025), to measure nutrient cycling rates and plant growth outcomes of the ecosystem services (Wang et al., 2023).

## 5 Conclusion

The application of L-GLA changed the soil microorganism communities and Carbon cycle metabolic functions. Specifically, L-GLA application significantly reduced the content of SOM and AK. Two doses of L-GLA treatment significantly changed soil microbial community diversity, structure and composition, and interfered with the microbial co-occurrence networks and metabolic functions. Based on Shannon and Simpson index, compared with the control, the recommended dose of L-GLA increased the community diversity, H treatment promoted the community diversity on 30-days post-application while decreased on 60 days after application. L-GLA treatments significantly increased the relative abundance of Actinomycetota. What’s more, L-GLA addition altered soil metabolic profile, interfered with the metabolic pathways involved by the characteristic metabolites and these alterations significantly associated with shifts in the differential microbial species. This research verified the intimate correlation between soil microbes and soil metabolism, which may provide a novel insight into ecotoxicological evaluation and risk assessment for further making highly effective environmentally friendly herbicides in the future leading a step towards sustainable agriculture.

## Data Availability

All data generated or analyzed during this study are included in this research article and its supplementary information files. Raw data of high-throughput sequencing of samples were deposited in the NCBI Sequence Read Archive (SRA) under the accession number PRJNA1173394.

## Footnotes

1
https://github.com/cjfields/readfq

2
http://topaz.gatech.edu/GeneMark/

3
https://github.com/bbuchfink/diamond/

4
http://www.kegg.jp/kegg/

5
https://www.metaboanalyst.ca

## Supplementary material

SUPPLEMENTARY FIGURE S1 Degradation of L-glufosinate in soil samples at different application rates **(A)**: application at 600 g a.i. hm^−2^, **(B)**: application at 3000 g a.i. hm^−2^.

SUPPLEMENTARY FIGURE S2 Effects of L-glufosinate-ammonium on microbial community structure. Principal co-ordinates analysis (PCoA) of the soil community based on Bray-Curtis distances on day 30 **(A)** and 60 **(B)**. CK: without L-glufosinate-ammonium addition; L: application at 600 g a.i. hm^−2^; H: application at 3,000 g a.i. hm^−2^.

SUPPLEMENTARY FIGURE S3 Relative abundances histograms of microbial community (top 40) at the genus level on day 30 **(A)** and 60-post application **(B)**, respectively. CK: without L-glufosinate-ammonium addition; L: application at 600 g a.i. hm^−2^; H: application at 3,000 g a.i. hm^−2^.

SUPPLEMENTARY FIGURE S4 Differential genus between CK and treatments at 30 and 60-post application, respectively. **(A,D)**: differential genera between CK and L on day 30 and 60, respectively; **(B,C)**: differential genera between CK and H on day 30 and 60, respectively. CK: without L-glufosinate-ammonium addition; L: application at 600 g a.i. hm^−2^; H: application at 3,000 g a.i. hm^−2^.

SUPPLEMENTARY FIGURE S5 Characteristic metabolic pathways based on KEGG database at level 3. **(A,B)**: differential metabolic pathways between CK and L on day 30 and 60, respectively; **(C,D)**: differential metabolic pathways between CK and H on day 30 and 60, respectively. CK: without L-glufosinate-ammonium addition; L: application at 600 g a.i. hm^−2^; H: application at 3,000 g a.i. hm^−2^.

SUPPLEMENTARY FIGURE S6 Influence of L-glufosinate-ammonium on soil carbon cycle-related enzymes activities. **(A)**: sucrase; **(B)**: catalase; **(C)**: soil amylase; **(D)**: polyphenol oxidase; **(E)**: β-glucosidase; **(F)**: N-acetyl-β-D-glucosidase. Lowercase letters (a, b, c) indicate significant differences among different treatments for a given time point using one-way ANOVA (*p* < 0.05). CK: without L-glufosinate-ammonium addition; L: application at 600 g a.i. hm^−2^; H: application at 3,000 g a.i. hm^−2^.

SUPPLEMENTARY FIGURE S7 Metabolic difference analysis of the L-glufosinate-ammonium treatments via principal component analysis at 30 d **(A)** and 60 d **(B)**, volcano plots of different metabolites between CK and L at 30 d **(C)** and 60 d **(D)**, and between CK and H at 30 d **(E)** and 60 d **(F)**. CK: without L-glufosinate-ammonium addition; L: application at 600 g a.i. hm^−2^; H: application at 3,000 g a.i. hm^−2^.

SUPPLEMENTARY FIGURE S8 Heatmap clustering of differential metabolites (Fold change value >2.0 and false discovery rate *p*-value <0.05) that were significantly affected by L-glufosinate-ammonium stress. **(A,C)**: differential metabolites between CK and L on day 30 and 60, respectively; **(B,D)**: differential metabolites between CK and H on day 30 and 60, respectively. CK: without L-glufosinate-ammonium addition; L: application at 600 g a.i. hm^−2^; H: application at 3000 g a.i. hm^−2^.

SUPPLEMENTARY TABLE S1 Physicochemical characteristics of tobacco soil under different treatments.

SUPPLEMENTARY TABLE S2 Topological parameters of soil microbial correlation interactions network.
